# Glucocorticoid receptor controls atopic dermatitis inflammation via functional interactions with P63 and autocrine signaling in epidermal keratinocytes

**DOI:** 10.1038/s41419-024-06926-w

**Published:** 2024-07-28

**Authors:** Lisa M. Sevilla, Omar Pons-Alonso, Andrea Gallego, Mikel Azkargorta, Félix Elortza, Paloma Pérez

**Affiliations:** 1 Instituto de Biomedicina de Valencia (IBV-CSIC), Department of Pathology and Molecular and Cell Therapy, Valencia, Spain; 2 Proteomics Platform, CIC bioGUNE, Basque Research and Technology Alliance (BRTA), CIBERehd, Science and Technology Park of Bizkaia, Derio, Spain

**Keywords:** Chronic inflammation, Endocrinology

## Abstract

Atopic dermatitis (AD), a prevalent chronic inflammatory disease with multifactorial etiology, features epidermal barrier defects and immune overactivation. Synthetic glucocorticoids (GCs) are widely prescribed for treating AD due to their anti-inflammatory actions; however, mechanisms are incompletely understood. Defective local GC signaling due to decreased production of endogenous ligand and/or GC receptor (GR) levels was reported in prevalent inflammatory skin disorders; whether this is a consequence or contributing factor to AD pathology is unclear. To identify the chromatin-bound cell-type-specific GR protein interactome in keratinocytes, we used rapid immunoprecipitation of endogenous proteins and mass spectrometry identifying 145 interactors that increased upon dexamethasone treatment. GR-interacting proteins were enriched in p53/p63 signaling, including epidermal transcription factors with critical roles in AD pathology. Previous analyses indicating mirrored AD-like phenotypes between P63 overexpression and GR loss in epidermis, and our data show an intricate relationship between these transcription factors in human keratinocytes, identifying *TP63* as a direct GR target. Dexamethasone treatment counteracted transcriptional up-regulation of inflammatory markers by IL4/IL13, known to mimic AD, causing opposite shifts in GR and P63 genomic binding. Indeed, IL4/IL13 decreased GR and increased P63 levels in cultured keratinocytes and human epidermal equivalents (HEE), consistent with GR down-regulation and increased P63 expression in AD lesions *vs* normal skin. Moreover, GR knockdown (GR^KD^) resulted in constitutive increases in P63, phospho-P38 and *S100A9*, *IL6*, and *IL33*. Also, GR^KD^ culture supernatants showed increased autocrine production of TH2-/TH1-/TH17-TH22-associated factors including IL4, CXCL10, CXCL11, and CXCL8. GR^KD^ HEEs showed AD-like features including hyperplasia and abnormal differentiation, resembling phenotypes observed with GR antagonist or IL4/IL13 treatment. The simultaneous GR/P63 knockdown partially reversed constitutive up-regulation of inflammatory genes in GR^KD^. In summary, our data support a causative role for GR loss in AD pathogenesis via functional interactions with P63 and autocrine signaling in epidermal keratinocytes.

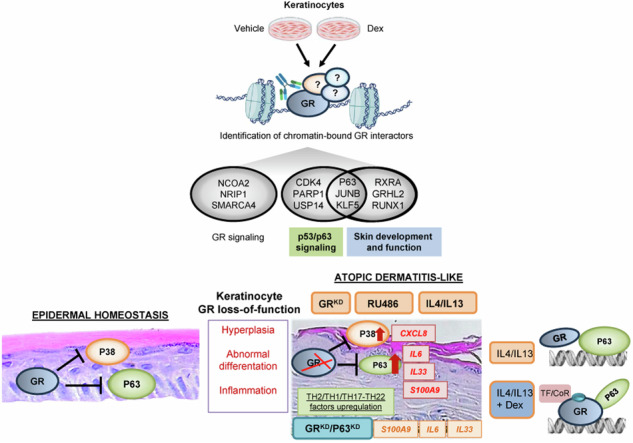

## Introduction

Atopic dermatitis (AD) is a prevalent chronic relapsing inflammatory disease with multifactorial etiology, characterized by epidermal barrier defects and immune overactivation, with predominant TH2-associated cytokine responses [1]. While the underlying mechanisms of AD are incompletely defined, the disease involves bidirectional interplays between keratinocytes and the skin immune system that leads to a vicious cycle of repetitive inflammation and impairment of barrier function [2].

Synthetic GCs remain the mainstay treatment for AD due to their effectiveness in reducing type 2 cytokines in patient’s serum and skin samples [3]; unfortunately, long treatments and/or high doses result in adverse effects including metabolic anomalies [4, 5]. Importantly, while GCs are produced in the adrenal glands and delivered to all organs systemically, the skin is also capable of de novo GC synthesis and therefore constitutes a biologically relevant extra-adrenal source allowing for rapid tissue-specific responses [6, 7]. However, the contribution of keratinocytes to GC-mediated immunoregulation has received limited attention [5, 8, 9].

GC actions are mainly mediated by binding and activation of the GC receptor (GR/NR3C1), an ubiquitous transcription factor (TF) vital for whole-body homeostasis, energy balance and stress response [10]. One major open question is to understand the exact mechanisms by which GR coordinates the transcription of thousands of genes in tissue- and cell-type-specific manners. The formation of distinct GR-containing oligomers, the composition and kinetics of GR genomic binding, and the interactions with cell-type-specific TFs and coregulators, among others, are crucial for the transcriptional outcome in response to GCs [11, 12].

Recently, we addressed a comprehensive analysis of GR genomic actions in keratinocytes by transcriptomic and cistromic approaches in immortalized wild-type mouse keratinocytes upon treatment with the synthetic GC ligand dexamethasone (Dex) [13]. GR ChIP-seq identified overrepresentation of motifs for GREs, AP-1, TEAD, and p63 [13]. However, the composition of protein complexes at genomic GR binding sites in keratinocytes, and the functional consequences of these interactions, remained unknown.

Previous studies in mice demonstrated that GR is required for skin barrier competence and control of inflammation as shown by the perinatal phenotype of defective keratinocyte differentiation and upregulation of inflammatory genes upon epidermal loss, overall resembling AD features [14]. Also, defective GC signaling, due to decreased ligand production and/or GR levels, has been reported in the skin of patients or mouse models with inflammatory skin disease [14–17]; however, whether this is a consequence of or a contributing factor to pathology is unclear.

In this study, we integrated diverse experimental approaches, including the Rapid immunoprecipitation mass spectrometry of endogenous protein (RIME) technique [18], chromatin binding and gene expression studies, identification of keratinocyte-specific secreted factors, and functional assays in keratinocyte monolayer and human epidermal equivalent (HEE) AD models, to gain understanding on the cell-type-specific role of GR in this disease.

We identified a cohort of chromatin-bound GR-interacting proteins, including TFs with critical roles in keratinocytes and AD pathology such as the epidermal lineage TF p63 [19]. In addition to being identified as a direct negative regulator of P63, ligand-activated GR counteracted transcriptional up-regulation of inflammatory markers in response to the TH2-associated cytokines IL4/IL13, known to mimic AD, by mechanisms that involve opposite GR and P63 genomic binding patterns. GR loss-of-function achieved via stable GR knockdown (GR^KD^) in primary human keratinocytes resulted in AD-like features of HEEs including hyperplasia and abnormal differentiation, constitutive increases in P63, upregulation of disease markers, and increased autocrine production of TH2-/TH1-/TH17-TH22-associated factors, relative to controls. Moreover, the simultaneous knockdown of GR and P63 partially reversed constitutive upregulation of key inflammatory genes in GR^KD^. In summary, our data support a causative role for GR loss in AD pathogenesis via functional interactions with P63 and autocrine signaling in epidermal keratinocytes.

## Results

### Protein interaction landscape of dexamethasone (Dex)-bound GR in keratinocytes

**Fig. 1:**
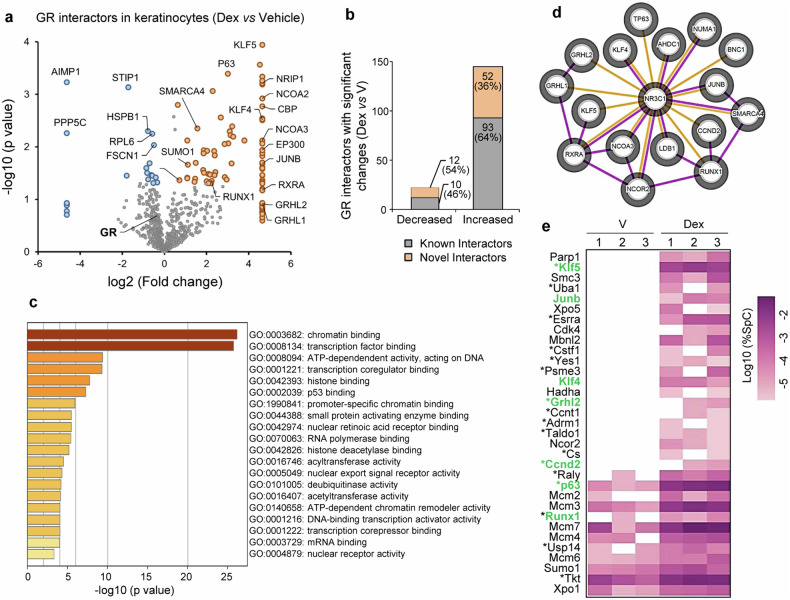
Identification of chromatin-bound GR-interacting proteins in dexamethasone (Dex)-treated keratinocytes. Mouse keratinocytes were treated with Dex (100 nM) or vehicle (V) for 1 h, and GR-bound chromatin fragments isolated for mass spectrometry. **a** Volcano plot shows GR interactors; loss (blue) or gain (orange) of interactions upon Dex treatment are indicated. Note that for visualization purposes, fold changes that could not be computed (present in one group and completely absent in the other) were attributed the maximum value. **b** The graph shows significantly changed GR interactions in Dex *vs* vehicle-treated cells. **c** Functional categorization of 145 significant Dex-induced interactors (Metascape, Molecular Function). **d** Network of GR-interacting proteins involved in skin/keratinocyte function (identified by IPA) was built using the STRING database using confidence level of 0.4 (default setting) and with no additional interactors. The orange edges represent novel GR interactors, while the purple edges represent previously reported interactions and/or predicted protein-protein networks (from databases or experimentally determined). **e** Heatmap of GR interacting proteins involved in p53/p63 signaling (IPA).

GR-containing protein complexes bound to chromatin were immunoprecipitated from immortalized mouse keratinocytes incubated in the absence (vehicle) or presence of the synthetic GC Dex (100 nM, 1 h) to identify the cell-type-specific protein interaction network. After processing, chromatin immunoprecipitates were digested and analyzed by mass spectrometry following the RIME technique [18]. We identified 811 protein interactors of GR (Table S1), and focused on those whose interactions were modulated by Dex treatment (Fig. 1a). Differences between Dex- and vehicle-treated cells were denoted as significant when proteins were found in 2 out of 3 replicates in one condition with an associated *p* value < 0.05 or in 2 out of 3 replicates from one group and completely absent in the other (Table S1). While Dex treatment resulted in a majority of gained interactions with GR, it elicited decreased association of proteins such as STIP1 and PPP5c (Fig. 1b; 87% and 13%, respectively).

For further analyses, only GR associated proteins with gain of interaction and significant enrichment upon treatment were considered (fold change Dex *vs* Veh >1.5). Functional categorization of these 145 GR-associated proteins showed enrichment for transcription factors (35%); chromatin binding proteins (28%); acetyltransferases (11%); histones (10%); nuclear receptor activity (9%); transcription coregulators (8%); p53 binding (6%); and histone deacetylases (5%) (Fig. 1c and Table S2).

Dex-induced interactors included previously known GR coregulators such as CBP, EP300, NCOA2, NCOA3, NCOR2, NRIP1, and SUMO1, as well as proteins involved in chromatin remodeling including different subunits of the BAF (SWI/SNF) complex such as ARID1A, SMARCA4, SMARCA5, and SMARCD2 (Table S2; [20]). Importantly, we also identified 52 GR novel interactors, including P63, Krüppel-like factor (KLF)5, RUNX1 Grainyhead-like (GRHL)1, and GRHL2 (Table S1). Ingenuity Pathway analyses (IPA) confirmed a network of GR-interacting proteins involved in skin/keratinocyte function (Fig. 1d; orange and purple edges indicate novel and previously reported interactors, respectively). Also, IPA identified enrichment in p53/p63 signaling in our dataset, including 51% novel GR interactors (Fig. 1e; asterisks) and 21% proteins specifically involved in skin/keratinocyte biology (Fig. 1e; in green). Importantly, most of these proteins have been related to skin inflammatory diseases and, in particular, to AD [21–24]. The fact that p63, the epithelial homolog of p53, appears as upstream regulator of numerous novel GR interactors suggested that this protein is central in the transcriptional hub involved in GR-dependent transcription in keratinocytes.

### GR/P63 interaction in keratinocytes

**Fig. 2:**
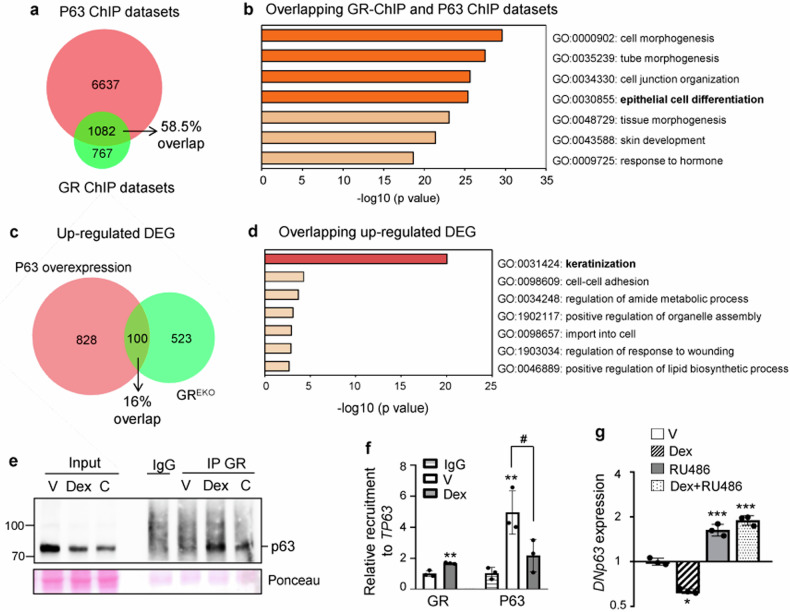
GR/p63 interaction in keratinocytes. **a** Venn diagram of ChIP-seq datasets for P63 bound to active enhancers (human) and GR (mouse). Note that almost 60% of GR bound genes are also bound by P63. **b** Functional categorization of overlapping GR and P63 bound genes in (**a**) for biological process with Metascape. **c** Venn diagram of up-regulated differentially expressed genes (DEG) in GR epidermal KO (GR^EKO^) epidermis and p63-overexpressing epidermis. **d** Functional categorization of overlapping genes in (c) with Metascape, biological process. **e** p63 immunoblot of nuclear GR immunoprecipitations (IP) in mouse keratinocytes treated 1 h with vehicle (V), 100 nM dexamethasone (Dex), or 100 nM corticosterone (C). **f** Chromatin immunoprecipitation shows Dex-dependent modulation of GR and P63 recruitment to *TP63* in N/TERT-2G (Dex, 1 μM, 1 h). **g** RT-QPCR for *DNP63* evaluating response to Dex (1 μM, 3 h) in N/TERT-2G that were pre-incubated 16 h with V or 10μM RU486. Statistical significance using Student’s t-test (**f**, GR) or one-way ANOVA (**f**, P63; **g**) with post hoc Tukey multiple comparison test: *^, #^*p* < 0.05; **p < 0.01; ***p < 0.001. Asterisks: significant differences relative to vehicle (**f**, GR; **g**) or IgG (**f**, P63); hash: significant differences between groups indicated by brackets. *N* = 3 for all experiments.

In silico analyses of ChIP-Seq datasets for GR (mouse) and P63 (human) in keratinocytes revealed that almost 60% of GR bound genes are also bound by P63 (Fig. 2a; [13, 25]). Categorization of this gene subset identified epithelial differentiation among the most overrepresented processes (Fig. 2b). Moreover, there was 16% overlap between up-regulated genes in epidermis from GR epidermal KO (GR^EKO^) mice and those with epidermal overexpression of p63 (Fig. 2c; [14, 21]). This subset showed enrichment for keratinization, a process intimately linked to inflammation, and cell-cell adhesion, including *Krt16*, *Krt17*, *Krt6a*, *Krt6b*, as well as genes of the epidermal differentiation complex such as *S100a8/9* and *Sprrs* (Fig. 2d). Also, gene set enrichment analysis identified overlap between differentially expressed genes in GR^EKO^ epidermis with P63 ChIP-Seq datasets (Fig. S2).

The interaction between GR and P63 in Dex-treated mouse keratinocytes was validated by co-immunoprecipitation of nuclear extracts followed by immunoblotting (Fig. 2e). Also, GR/P63 interaction was further validated using the endogenous GC corticosterone (100 nM; 1 h) as ligand (Fig. 2e), confirming that the interaction can occur in physiological and pharmacological conditions.

To further assess the relationship between GR and P63 in human keratinocytes, we used the N/TERT-2G cell line [2]. Chromatin immunoprecipitation experiments showed that while 1 h Dex treatment increased GR binding, it reduced P63 binding to the same *TP63* intronic region by 2-fold (Fig. 2f) despite similar P63 protein levels at this timepoint (Fig. S3). At 3 h Dex inhibited *DNP63* expression in a GR-dependent manner, as shown by the lack of repression in the presence of the GR antagonist RU486 (Fig. 2g).

### GR and P63 show inverse correlation in IL4/IL13-treated keratinocytes

**Fig. 3:**
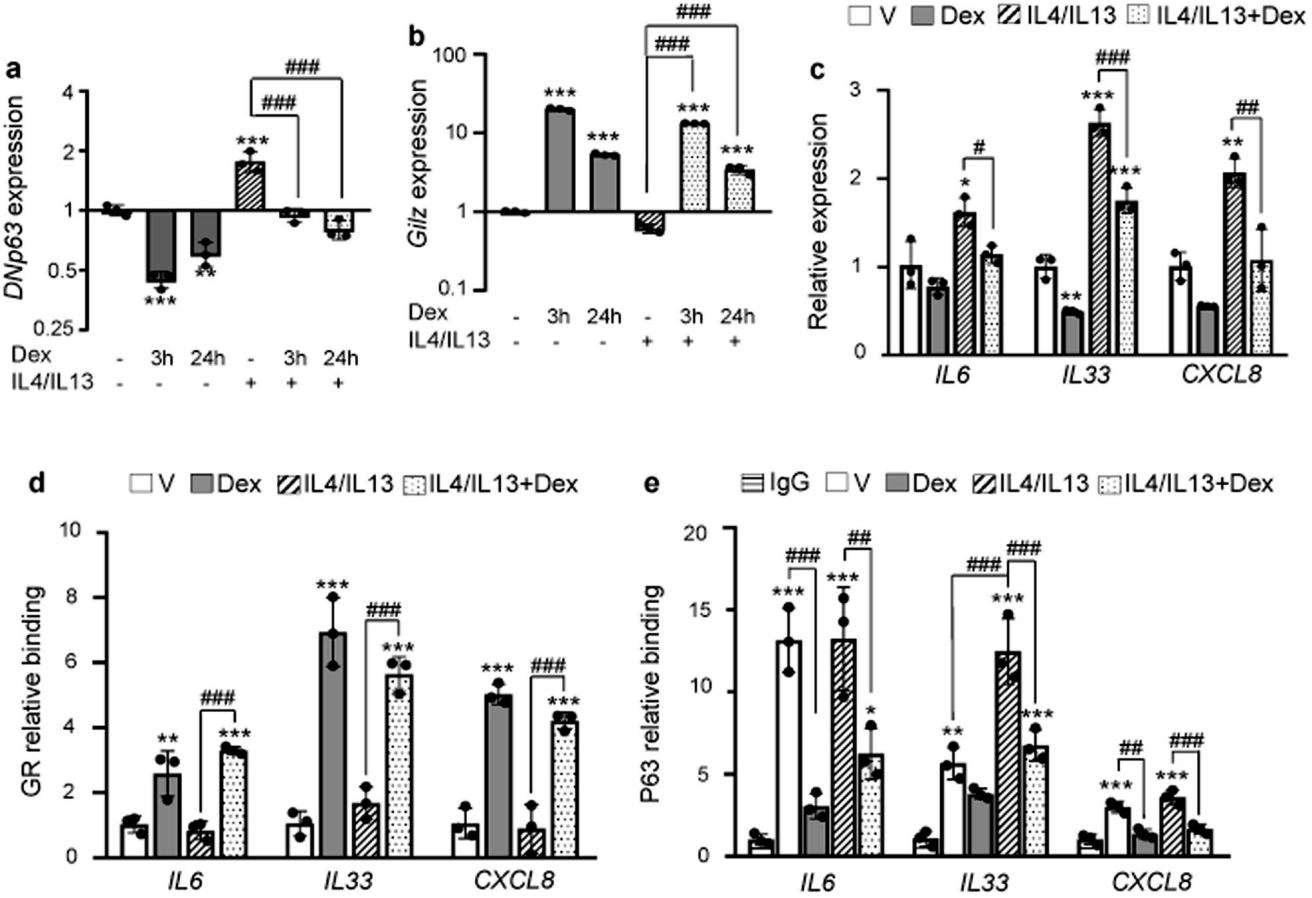
GR and P63 show inverse correlation in IL4/IL13-treated keratinocytes. Gene expression of *DNP63* (**a**) and *TSC22D3/GILZ* (**b**) after treatment with Dex (1 μM, 3 and 24 h), IL4/IL13 (50 ng/ml, 24 h), or both. **c** RT-QPCR evaluating indicated genes following 24 h incubation with vehicle (V), Dex (1 μM), and/or IL4/IL13 (50 ng/ml). ChIP-QPCR shows Dex (1 μM, 1 h) and IL4/IL13 (50 ng/mL, 16 h)-dependent modulation of GR (**d**) and P63 (**e**) recruitment to *IL6, IL33* and *CXCL8*. Statistical significance using one-way ANOVA with post hoc Tukey multiple comparison test: *^,^^#^*p* < 0.05; **^,^^##^*p* < 0.01; ***^, ###^*p* < 0.001. Asterisks: significant differences relative to vehicle (**a**–**d**) or IgG (**e**); hashes: significant differences between groups indicated by brackets. *N* = 3 for all experiments.

As p63 has a crucial role in epidermal homeostasis and AD pathogenesis [19, 26], we used N/TERT-2G cells treated with TH2 cytokines IL4/IL13 as an in vitro model of the disease [2, 27]. IL4/IL13 cytokines (50 ng/ml, 24 h) induced *TP63* but showed a trend towards inhibition of *TSC22D3/GILZ*, a classic GR-target gene with anti-inflammatory roles in most cell types (Fig. 3a, b). In turn, Dex exerted opposite effects on *TP63* and *TSC22D3/GILZ* expression relative to IL4/IL13; moreover, Dex was able to counteract IL4/IL13 effects on gene expression (Fig. 3a, b). Importantly, cytokine treatment also induced the expression of the inflammatory markers *IL6*, *IL33*, and *IL8*/*CXCL8*, which are P63 targets; notably, combined treatment of Dex plus cytokines reduced the induction of these pro-inflammatory genes (Fig. 3c).

To understand the mechanisms underlying gene repression, we assessed recruitment of GR and P63 to genomic regulatory regions identified in a P63 ChIP-seq [25] that also contain GREs. While Dex increased GR binding to *IL6*, *IL33*, and *CXCL8*, it decreased P63 binding to *IL6* and *CXCL8*, with a trend towards reduced recruitment to *IL33* (Fig. 3d, e). IL4/IL13 treatment increased P63 recruitment only to *IL33* but, notably, the presence of both cytokines and Dex resulted in increased and decreased recruitment of GR and P63, respectively, relative to cytokines alone (Fig. 3d, e), consistent with the represion of these inflammatory targets (Fig. 3c).

**Fig. 4:**
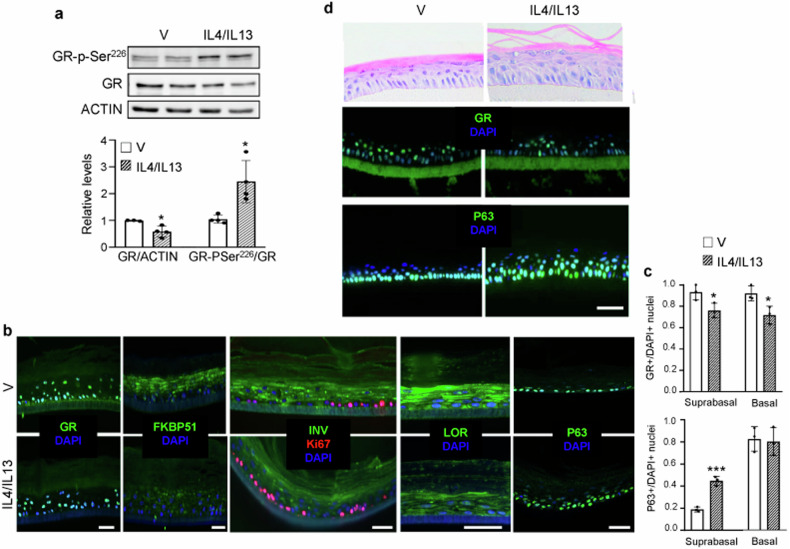
IL4/IL13 treatment results in reduction of GR expression and function in human keratinocytes. **a** Primary human keratinocytes were treated 72 h with vehicle or 30 ng/ml IL4/IL13 prior to immunoblotting (upper); quantitation (lower). *N* = 4. **b** Immunofluorescence of sections of human epidermal equivalents (HEEs) with primary keratinocytes treated with vehicle (V) or IL4/IL13 (30 ng/ml, 72 h) with indicated antibodies. **c** Graphs showing ratios of GR+ (top) or P63+ nuclei (bottom) relative to total DAPI stained nuclei in HEEs treated with vehicle (V) or IL4/IL13. Statistical significance determined using Student’s t-test, *p < 0.05; ***p < 0.001. *N* = 3. **d** Sections of HEEs with N/TERT-2G cells treated with vehicle (V) or IL4/IL13 (50 ng/ml, 72 h). Top panels: hematoxylin & eosin. Lower panels: immunofluorescence with indicated antibodies. Bars: 50 μm.

Importantly, treatment of primary human keratinocytes with IL4/IL13 (30 ng/ml, 72 h) resulted in a 40% reduction in GR protein levels together with 2-fold increase in phosphorylation of Ser^226^, which induces GR nuclear export (Fig. 4a). The effects of IL4/IL13 were recapitulated in human epidermal equivalents (HEEs), where GR expression decreased in both suprabasal and basal layers (Fig. 4b, c) together with significant down-regulation of its downstream target FKBP51 (Fig. 4b). This partial GR-loss-of-function was consistent with augmented proliferation and impaired differentiation in IL4/IL13-treated HEEs, as shown by an increase in Ki67-positive nuclei and a decrease in Involucrin and Loricrin (Fig. 4b). Moreover, changes correlated with augmented P63 staining and 2.4-fold increased P63-positive suprabasal nuclei in IL4/IL13- *vs* vehicle-treated HEEs (Fig. 4b, c). These results were recapitulated in HEEs generated from immortalized N/TERT-2G keratinocytes (Fig. 4d).

### GR loss-of-function induces inflammation and AD-like phenotypic features that can be partially reversed by simultaneous knockdown of P63

**Fig. 5:**
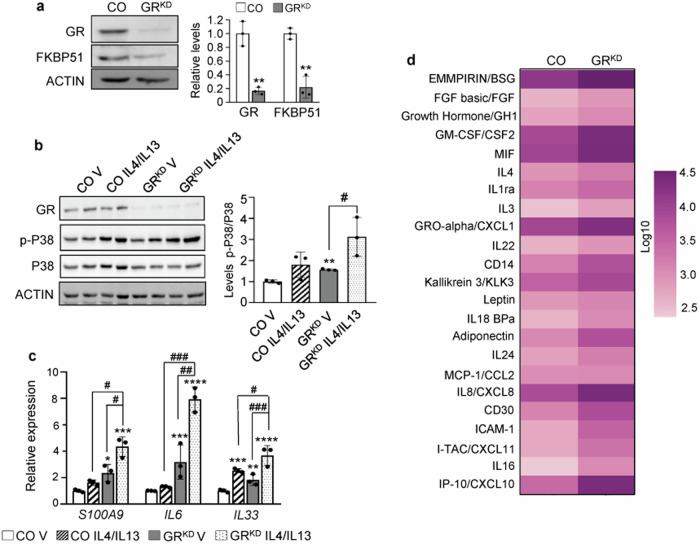
Keratinocyte GR loss-of-function results in enhanced inflammation in human keratinocytes. **a** Immunoblot (left) and quantitation (right) of control (CO) or GR-knockdown (GR^KD^) keratinocyte lysates with indicated antibodies. **b***,* **c** CO or GR^KD^ keratinocytes were treated 24 h with vehicle (V) or 50 ng/ml IL4/IL13. Immunoblotting (**b**) with P38 and phospho(p)-P38 antibodies (left); quantitation (right). RT-QPCR (**c**) for expression of indicated genes. Mean and SD are shown in graphs. Statistical significance using Student’s t-test (**a**) or 2-way ANOVA and post hoc Tukey multiple comparison test (**b**, **c**) indicated as: *^,^^#^*p* < 0.05; **^, ##^*p* < 0.01; ***^, ###^*p* < 0.001. Asterisks: statistically significant differences relative to CO V; hashes: comparisons between groups indicated by brackets. *N* = 3 for all experiments. **d** Heatmap shows relative levels of secreted factors in CO or GR^KD^ primary keratinocyte supernatants (vehicle-treated) using a multiplex antibody array. Data are average of three independent experiments and represent factors with statistical significance using Student’s *t*-test; *p* < 0.05.

To further assess whether GR loss-of-function in human keratinocytes has a causal role in AD or increases susceptibility to TH2 cytokines, we generated stable knockdown of GR in primary keratinocytes (GR^KD^) via infections with shRNA-containing lentivirus (Fig. 5a). GR^KD^ keratinocytes showed 80% reduction in GR and a 70% reduction in FKBP51 relative to those expressing scrambled shRNA (control; CO; Fig. 5a). In GR^KD^ keratinocytes, P38 phosphorylation was basally augmented, and further increased upon treatment with IL4/IL13 (Fig. 5b). Also, *S100A9*, *IL6*, and *IL33* levels were constitutively increased in GR^KD^ *vs* CO; and further increased after cytokine treatment (Fig. 5c). Moreover, intracellular S100A9 and IL33 protein levels were basally augmented in GR^KD^ *vs* CO cells, with a trend towards IL6 up-regulation (Fig. S4).

To assess the profile of factors secreted by GR^KD^ keratinocytes, we performed antibody arrays using conditioned media from monolayer cultures. Basally, GR^KD^ showed strong and significant up-regulation of 23 inflammatory markers with known roles in AD (Fig. 5d*p* < 0.05; and Table S3 [28]). Up-regulated factors showed a merged profile of TH2-/TH1-/TH17-TH22-associated markers including GM-CSF/CSF2, IL1RN, IL4 (TH2); IP-10/CXCL10; I-TAC/CXCL11, IL16 (TH1); and IL8/CXCL8, GRO alpha/CXCL1, IL22 (TH17-TH22); Fig. 5d). This profile was consistent with the reported up-regulation of inflammatory markers in the epidermis of AD lesioned skin as well as a shift from early TH2 profile towards a TH1/TH17-TH22 response in chronic AD [29, 30]. Upon IL4/IL13 treatment, 35% of constitutively upregulated factors showed further increase; also, MIP3alpha/CCL20, which was unchanged in basal conditions, showed fourfold higher levels in GR^KD^ *vs* CO keratinocytes (Fig. S5 and Table S3).

**Fig. 6:**
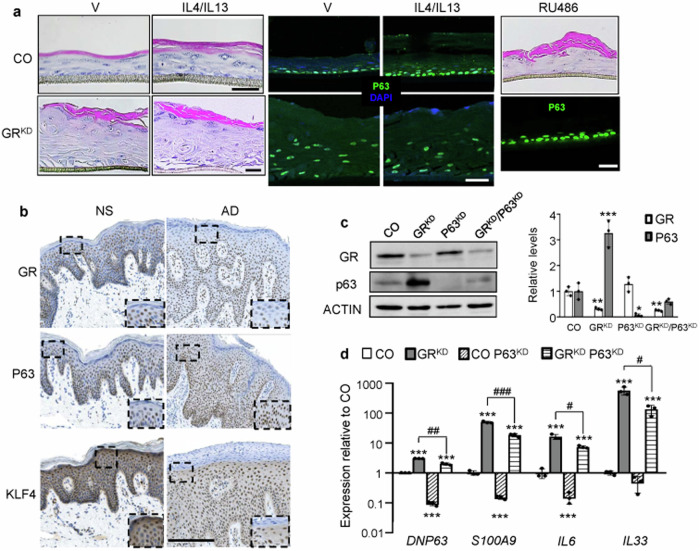
Atopic dermatitis inflammation upon GR loss-of-function can be partially rescued by simultaneous GR/P63 knockdown. **a** Sections of GR-knockdown (GR^KD^) and control (CO) HEEs treated with vehicle (V) or 50 ng/ml IL4/IL13 (72 h) or 5μM RU486 (13 d) stained with hematoxylin & eosin (left panels and upper panel RU486) or P63-specific antibodies. Bars: 50 μm. *N* = 3. **b** Sections from normal (NS) or lesional (AD) skin. *N* = 2 for each condition. Bar: 300 μm. **c**, **d** CO or GR^KD^ N/TERT-2G were transiently transfected with negative control or P63-specific (P63^KD^) siRNAs. Western blot (left) and quantification (right) show knockdown of GR and P63 (**c**). RT-QPCR for indicated genes (**d**). *N* = 3. Mean and SD are shown in graphs. Statistical significance using 2-way ANOVA (**d**) with post hoc Tukey multiple comparison test: *^, #^*p* < 0.05; **^, ##^*p* < 0.01; ***^, ###^*p* < 0.001. Asterisks: significant differences relative to CO; hashes: significant differences between groups indicated by brackets (**d**).

GR^KD^ HEEs showed a basal phenotype of hyperplasia and abnormal differentiation similar to that elicited by IL4/IL13 treatment in CO (Fig. 6a). However, cytokine exposure did not trigger further phenotypic changes in GR^KD^ equivalents (Fig. 6a). Importantly, pharmacological blockade of GR with RU486 during air-liquid interface culture also caused hyperplasia and accumulation of cornified layers (Fig. 6a). Also, phenotypic alterations of GR^KD^ and RU486-exposed CO HEEs correlated with increased P63 staining similar to those treated with IL4/IL13 (Figs. 6a and 4b). Changes strongly resembled alterations induced by overexpression of P63 in mouse models as well as human AD lesions [19, 21, 26]. Indeed, immunostaining of AD skin lesions revealed increased expression of P63 together with down-regulation of GR and KLF4, relative to normal skin (Fig. 6b).

Next, we assessed whether alterations in gene expression in GR^KD^ could be reversed by knockdown of P63. N/TERT-2G stably expressing scrambled (CO) or GR specific shRNAs (GR^KD^) were transiently transfected with negative control or P63-specific (P63^KD^) siRNAs (Fig. 6c). CO keratinocytes with P63^KD^ showed significantly down-regulated levels of *S100A9* and *IL6*, consistent with the fact that they are downstream P63-target genes [19]; Fig. 6d). Importantly, while expression of these inflammatory genes along with *IL33* was constitutively increased in GR^KD^ *vs* CO cells, significant gene down-regulation was observed in GR^KD^/P63^KD^ relative to GR^KD^ keratinocytes (Fig. 6d).

In contrast to the above mentioned inflammatory genes, and although *CXCL8* was down- and up- regulated in P63^KD^ and GR^KD^ cells, respectively, its expression was not decreased in GR^KD^/P63^KD^ *vs* GR^KD^ keratinocytes (Fig. S6a). As P38 was reported to control P63-dependent expression of pro-inflammatory genes [31], we assessed the effects of P38 inhibitor BIRB796 (1 μM, 24 h) on *CXCL8* expression in GR^KD^ keratinocytes and found twofold reduction in levels of this chemokine (Fig. S6b). However, other inflammatory genes such as *IL6* and *S100A9* showed only a trend towards reduced levels upon P38 inhibition (data not shown). Overall, data show mutual GR/P63 interference and opposite transcriptional regulation of key inflammatory genes and indicate that P63 downstream effects are both P38-dependent and -independent.

## Discussion

GCs are the most widely used therapeutic agents for treating chronic inflammatory conditions, due to the effective anti-inflammatory role of GR in multiple cell types. While GR is able to regulate ∼20% of the human genome [9], the transcriptional response to GCs is highly dependent on the cell-type and relies on protein-protein interactions between GR and specific TFs and coregulators [11, 12]. To date, only few studies investigated the molecular composition of the GR transcriptional complex by RIME, including a recent study identifying interactors in human lung cancer cell lines [32]. Our data represent, to the best of our knowledge, the first report of the GR protein interaction network on chromatin in keratinocytes, with the added value of having identified interactions between endogenous proteins. The presence of known GR interactors including coregulators that are critical for GR-driven transcriptional regulation such as CBP, EP300, and NCOA/NCOR family members, technically validates our RIME analyses [20]. Importantly, the identification of novel GR interactors with major roles in regulating the proliferation/differentiation balance in keratinocytes such as P63, KLF5, RUNX1, GRHLl, and GRHL2 [33] (Fig. S1) suggests a hub for transcriptional control in keratinocytes in response to GCs, with relevance in epidermal homeostasis. These findings are consistent with the overrepresentation of p53/p63, KLF, and RUNX motifs in previous GR ChIP-seq studies [13] and data not shown]. We previously demonstrated transcriptional cooperation between GR and Klf4 in regulating gene expression in keratinocytes [34–36]. As DNP63 inhibits *Klf4* via direct binding to its promoter [37], P63 up-regulation upon GR loss-of-function may contribute to *Klf4* repression. This is consistent with the inverse correlation between the P63 expression and that of GR and KLF4 immunostaining pattern in the epidermis of AD lesional skin (Fig. 6b). Altogether, data indicate that GR is involved in multiple regulatory loops aimed to provide flexibility in the GC transcriptional response under different physiopathological settings [5], and warrant additional studies of other identified novel partners.

Previous phenotypic analyses were suggestive of a functional relationship between GR and the master transcriptional regulator P63. Heterozygous mutations in *TP63* cause rare ectodermal dysplasia disorders and mouse models with either knockout of DNp63 or epidermal overexpression of GR show similar phenotypes with characteristics of these syndromes [38–40]. The global knockout of p63 is lethal due to the absence of the epidermis, indicating that it has a central role in establishing epithelial cell fate [41, 42]. Global GR knockout mice also died a few hours after birth due to respiratory problems and featured defective skin barrier function due to the lack of stratum corneum [34]. Importantly, phenotypes upon epidermal loss of GR mirrored those upon overexpression of DNp63, the more abundant p63 isoform in epidermis [14, 19, 21]. AD-like features in GR^EKO^ epidermis included up-regulation of inflammatory genes that are known p63 targets and markers of the disease; however, the underlying mechanisms were unknown [14].

The marked overlap between GR- and P63- genomic binding as well as gene expression profiles, with enrichment in keratinocyte differentiation (Fig. 2a–d), together with the unbiased identification of p53/p63 signaling enrichment among the GR-associated proteins in keratinocytes supports a role for the GR/p63 network in epidermis. Our data show an intricate relationship between these TFs, which includes several layers of regulation. Dex treatment increased interaction between GR and p63 (Fig. 1e), but also inhibited *DNP63* expression (Fig. 2g), revealing a feedback loop intended to limit P63-dependent actions in keratinocytes. Moreover, Dex elicited opposite GR and P63 binding patterns to inflammatory genes, alone or in combination with cytokines, suggesting that this may represent a general mechanism underlying gene repression (Figs. 2f, g and 3c–e). This inverse binding pattern may be attributed to several non-exclusive mechanisms, including competition for limited quantities of coregulators (squelching) and P63 displacement from chromatin due to alternative interactions between GR and other TFs/coregulators, which ultimately disrupt P63-dependent transcription. Indeed, several proteins identified in this study were also p63 interactors, including SMARC4/BRG1, EP300 and SUMO1 [43]. P63 has been reported to regulate the chromatin landscape by directly transcribing chromatin-modulating factors such as SMARC4/BRG1, which allows nucleosome remodeling at the epidermal differentiation complex, a keratinocyte lineage-specific gene cluster [43]. Also, the increased GR/P63 interaction upon Dex may elicit both DNA binding –independent (tethering) and –dependent (direct binding of GR to genomic sites near P63) mechanisms to repress inflammatory genes, similar to that reported for GR/NF-κB antagonism [44–46].

One important finding is that GR loss-of-function results in AD-like features including abnormal keratinocyte proliferation and differentiation, and basal enhanced inflammation (Fig. 6). The opposite effects of IL4/IL13 on P63 and GR expression, together with the up-regulation of P63 upon GR loss-of-function via knockdown or pharmacological antagonism indicates negative interference between these TFs. Further, the fact that P63 knockdown partially rescued the constitutive increases of expression of inflammatory genes *S100A9*, *IL6* and *IL33* provoked by GR knockdown, demonstrates the opposing roles of these TFs in regulating keratinocyte inflammatory signaling (Figs. 5c and 6d).

However, the up-regulation of *CXCL8*, which has key roles in AD and is strongly increased in the supernatants of GR^KD^ *vs* CO cells (Figs. 5d and S6a), was not counteracted by P63 knockdown, suggesting the involvement of additional factors in its regulation. P38 phosphorylation was increased upon GR^KD^ and previous studies identified a link between P38 signaling and P63 regulation of inflammatory genes, in particular *MMP13* [31]. However, P38 inhibition was only successful for reduction of *CXCL8* in GR^KD^ keratinocytes, having only marginal effects on *IL6*, *IL33*, and *S100A9* (Fig. S6b and data not shown), indicating that for these genes in this context, P63 actions occur independently of P38.

The uncontrolled inflammation upon keratinocyte GR loss is consistent with the critical role of local GC production in the regulation of epithelial immune microenvironment [16] and effectiveness of GC treatments in reducing type 2 cytokines in patient’s serum and skin samples [3]. Corticosteroids are known immunoregulators that exert effective therapeutic anti-inflammatory actions via GR by targeting multiple players of the type 2 immune response, including Th2 lymphocytes, mast cells, basophils, eosinophils, and antigen-presenting cells [8, 47]. Also, GCs contribute to restore the function of regulatory T cells, thereby controlling their defective activity associated to atopy [16].

Our findings emphasize the importance of keratinocytes as a major source of cytokines, chemokines, and growth factors that amplify the inflammatory response and contribute to AD pathogenesis, highlighting the strong immunomodulatory potential of keratinocyte GR in control of TH2/TH1/TH17 inflammatory mediators [1, 47]. In this regard, epidermal keratinocytes should be considered as cellular targets for disease prevention by breaking the vicious cycle of chronic inflammation [2]. Many of the identified factors constitutively up-regulated in GR^KD^ *vs* CO keratinocytes including IL4, IL8, IL24, CXCL10, CXCL11, and GM-CSF/CSF2 are overexpressed in AD skin (Fig. 5d and Table S3; [47, 48]). Also, many of them such as CCL2, CXCL8, CXCL10, and CXCL11, act as immune chemoattractants, resulting in further recruitment of T cells into inflamed skin. The fact that CCL20, a chemoattractant for TH17 and dendritic cells, showed more pronounced up-regulation by IL4/IL13 treatment in GR^KD^ *vs* CO cells, indicates that GR loss-of-function may exacerbate skin inflammatory responses [48].

In summary, we have identified the GR protein interaction network in keratinocytes upon GC treatment, which was enriched in p63 signaling and keratinocyte function. Our data indicate that GR is an upstream negative regulator of P63 in healthy and diseased keratinocytes, where both TFs play opposing roles. Our findings also support a novel causal role for GR in AD pathogenesis as GR loss-of-function due to knockdown on keratinocytes in monolayer and HEEs mimicked the phenotype and inflammatory profile of the disease. The identified GR/P63 interplay in keratinocytes opens the possibility of novel therapeutic strategies for cutaneous disorders where the combined use of lower GC doses together with P63 inhibitors may provide clinical benefit.

## Materials and methods

### Cell culture

All cell culture was carried out in ThermoFisher Forma (Eindhoven, Netherlands) 3121 incubators with 5% CO_2_ and 95% relative humidity. Immortalized mouse keratinocyte cell lines were generated from 8-wk-old female mouse dorsal skin [49] were cultured with mitomycin C (Cayman Chemical, Ann Arbor, MI)-treated J2-3T3 in type I collagen-coated flasks with FAD medium (calcium-free DMEM (Gibco, ThermoFisher) -Ham’s F12 (Biowest, Newry and Mourne, UK) (3:1) supplemented with 0.18 mM adenine (Sigma-Merck, Darmstadt, Germany), 0.35 mM calcium chloride (Sigma-Merck), 7.5% fetal calf serum (Biowest), 100 U/ml penicillin/100 μg/ml streptomycin (Capricorn Scientific, Ebsdorfergrund, Germany), 2 mM glutamine (Biowest), 0.25 μg/ml amphotericin B (Biowest), 5 μg/ml insulin (Capricorn Scientific), 0.1 nM cholera toxin (Sigma-Merck), and 10 ng/ml EGF (PeproTech, ThermoFisher). Primary human keratinocytes (CnT epidermal keratinocyte progenitors, juvenile, pooled; CELLnTEC) were cultured in CnT-Prime Epithelial Proliferation Medium (CELLnTEC, Bern, Switzerland). Generation of human epidermal equivalents (HEEs) was carried out as described [50]. Briefly, 3 × 10^5^ low passage keratinocytes were plated on collagen I (from rat tail; Gibco) -coated Falcon® (Corning Inc., Corning, NY) permeable inserts for 12-well plates with 0.4 μm translucent high density pores and grown for 48 h submerged in CnT-Prime media. Next, media was changed to a 70:30 mixture of Cnt-Prime 3D Barrier Media (CELLnTEC):DMEM (Biowest) and cells were incubated another 24 h in submerged culture. Keratinocytes were then cultured for 14 days at the air-liquid interface. For experiments in submerged culture, to mimic differentiating conditions present in 3D culture, cells were incubated for indicated times with 70:30 Cnt-Prime 3D Barrier Media:DMEM.

Recombinant human interleukins 4 and 13 (PeproTech) were added at indicated concentrations (30–50 ng/ml) to induce TH2 inflammation. The following reagents were used at the indicated concentrations: Dexamethasone (Dex; Sigma-Merck; 100 nM–1 μM), GR-antagonist RU486 (Sigma-Merck; 10μM), and BIRB796 (Tocris, Avon, UK; 1 μM).

N/TERT-2G immortalized human keratinocytes and related protocols were kindly provided by Natalie Jonca and Michel Simon, with the permission of James Rheinwald [51]. For submerged culture, cells were grown either in Epilife (Gibco) medium or with low-calcium (0.07 mM) FAD medium (3:1 Calcium-free DMEM: Hams F12 supplemented with 10% Chelex-100 50–100 mesh (Sigma-Merck)-treated FCS, 0.18 mM adenine, 5 μg/ml insulin, 0.1 nM cholera toxin, 10 ng/ml EGF, 100 U/ml penicillin/100 μg/ml streptomycin, 2 mM glutamine, 1.4 µM hydrocortisone (Sigma-Merck). To assess response to the synthetic glucocorticoid dexamethasone (Dex; Sigma-Merck), keratinocytes were pre-cultured 24 h in low-calcium FAD medium containing charcoal (Panreac, Darmstadt, Germany)-stripped serum or in FAD medium with 300 nM hydrocortisone. 24 h prior to experimental endpoint, media was supplemented with 1.5 mM calcium to stimulate differentiation.

For HEE culture, 3.5 × 10^5^ cells were plated in 12 mm diameter Millicell (Merck) polycarbonate 0.4 µm pore size cell culture inserts in Epilife medium supplemented with 1.5 mM calcium. 48 h later, cells were shifted to air-liquid interface culture with 1.5 mM calcium-containing Epilife supplemented with 92 μg/ml vitamin C (Sigma-Merck) and 10 ng/ml KGF (Peprotech) for 14 days.

J2-3T3 cells were generously provided by F. Watt’s laboratory. J2-3T3 cells were cultured in DMEM supplemented with 10% Bovine serum (Gibco), 100 U/ml penicillin/100 μg/ml streptomycin, 2 mM glutamine, and 0.25 μg/ml amphotericin B.

HEK293T cells (CRL-3216, ATCC) were cultured in DMEM supplemented with 10% FCS, 2 mM glutamine and 100 U/ml penicillin/100 μg/ml streptomycin.

### Rapid immunoprecipitation mass spectrometry of endogenous protein (RIME)

Unless otherwise mentioned all chemicals were from Sigma-Merck. Following hormone deprivation and treatment with vehicle (EtOH) or 100 nM Dex for 1 h, RIME experiments were performed as previously described [18]. Briefly, mouse keratinocytes (3 independent biological replicates per experimental group) were cross-linked with 1% formaldehyde solution, quenched with 0.1 M glycine and harvested in cold PBS. Nuclei were separated and sonicated (30 s on 30 s off cycles for 10 min) for chromatin fragmentation using a Bioruptor Pico (Diagenode, Liege, Belgium). Nuclear lysates were immunoprecipitated overnight with 6 μg GR antibody (Santa Cruz Biotechnology, Heidelberg, Germany; sc-1004) or non-specific rabbit IgG (Merck) precoupled to Protein A Dynabeads (Invitrogen, ThermoFisher; 10001D). The following day, the beads were washed 7 times with RIPA buffer (50 mM HEPES pH 7.6, 1 mM EDTA, 1% IGEPAL CA-630, 0.7% sodium deoxycholate, 0.5 M LiCl) and 3 times with 100 mM ammonium bicarbonate.

Chromatin immunoprecipitates were processed for Mass spectrometry analysis as previously described [18], with minor modifications. Briefly, beads were resuspended in 100 µl of 100 mM ammonium bicarbonate and trypsin was added (10 ng/µl) to an approximate enzyme-to-protein ratio of 1:100. Samples were allowed to digest overnight. Then, additional 10 µl of trypsin were added and samples were allowed to digest for 4 h. Samples were placed on a magnetic rack to collect the supernatant and recover peptides. Formic acid was added to a final concentration of 5% and peptides were desalted and resuspended in 0.1% formic acid using C18 stage tips (Millipore, Merck).

For MS analysis, samples were analyzed in a timsTOF Pro with PASEF mass spectrometer (Bruker Daltonics, Bremen, Germany) coupled online to an Evosep ONE liquid chromatograph (Evosep Biosystems, Odense, Denmark). 200 ng were directly loaded onto the Evosep ONE and resolved using the 30 samples-per-day protocol. timsTOF was operated in data-dependent mode using the standard 1.1 s cycle time acquisition method using default parameters. Protein identification and quantification was carried out using PEAKS X software (Bioinformatics Solutions, Waterloo, Canada). Searches were carried out against a database consisting of *Mus musculus* entries from Uniprot Swissprot (https://www.uniprot.org/). Precursor and fragment tolerances of 20 ppm and 0.05 Da were considered for the searches, respectively. Only proteins identified with at least one peptide at FDR < 1% were considered for further analysis and all proteins also identified in IgG negative control were excluded. Spectral counts (SpC) were normalized against the sum of SpC per sample, and percentages (% SpC) used for semiquantitative analysis of the data. Student’s t-test was applied to compare groups. Differences between Dex- and vehicle-treated cells (gain or loss in interactions) were denoted as significant if proteins were found in at least 2 out of 3 replicates in one condition with an associated *p* value < 0.05 or proteins were found in 2 of 3 replicates from one group and completely absent in the other.

For functional analysis, Metascape and Ingenuity pathway analysis was used to characterize the molecular events behind the differential protein patterns (Twist Bioscience, San Franscisco, CA). The calculated *p* values for the different analyses determine the probability that the association between proteins in the dataset and a given process, pathway or upstream regulator is explained by chance alone, based on a Fisher’s exact test (*P* value < 0.05 being considered significant).

### Compilation of previously reported GR interactors

Known GR-interacting proteins were compiled from previous publications [32, 52–54] and databases: BioGRID v4.4.229 (https://thebiogrid.org; RRID:SCR_007393) [55], STRING v12.0 (https://string-db.org; RRID:SCR_005223) [56], IID v2021-05 (http://ophid.utoronto.ca) [57], HuRI (http://interactome-atlas.org/; RRID:SCR_015670) [58] and comPPI v2.1.1 (https://comppi.linkgroup.hu/) [59]. For BioGRID, both human and mouse GR interactors were considered from all high and low throughput assays. For STRING and IID, both human and mouse GR interactors were considered from experimentally determined interactions; for STRING, only minimum medium confidence score of 0.4 was used. HuRI and comPPI is limited to human protein-protein interactions.

### Nuclear extraction and Co-immunoprecipitation

Nuclei (3 independent biological replicates per experimental group) were separated according to the RIME protocol and immunoprecipitated overnight with GR antibody or non-specific rabbit IgG preconjugated to Protein A Dynabeads (Invitrogen). The beads were washed 7 times with RIPA buffer and then boiled in Laemmli buffer and run on SDS-PAGE gels with appropriate input controls (3% of samples) prior to analysis by Western blotting.

### Chromatin immunoprecipitation

Peaks from a P63 ChIP-Seq in proximity to to TP63, IL6, IL33, and CXCL8, were evaluated for GR-response elements (GREs) and P63 motifs [25]. ChIP experiments were performed as previously described [35]. Briefly, following treatment with vehicle (EtOH) or 1 µM Dex for 1 h, and/or IL4/IL13 (50 ng/ml) for 16 h, keratinocytes (3 independent biological replicates per experimental group) were fixed with 1% formaldehyde for 8 min at room temperature, lysed and sonicated using a Bioruptor Pico set for ten 30 s ON/ 30 s OFF cycles to fragment chromatin. Input controls were 2% of chromatin used for ChIP. IP was done with antibodies indicated in Table S4 and Dynabeads™ Protein G or A magnetic beads (Invitrogen).

### Lentiviral infections

The bacterial glycerol stock containing the PKLO.1 lentiviral vector with the MISSION shRNA TRCN0000245007, specific to a region in the *NR3C1* 3′ UTR, was from Merck. The PKLO.1 scrambled shRNA negative control (#136035) and the second generation envelope pMD2.G (#12259) and packaging psPAX2 (#12260) plasmids were from Addgene (Watertown, MA). A GFP containing lentiviral vector (pLentiGFP) was used to control for efficiency of transfection and infection (Cell Biolabs, San Diego, CA). Lentiviral plasmids and polyethanolamine, linear 25,000 MW (Polysciences, Hirschberg, Germany) were added to Opti-MEM^TM^ (Gibco), vortexed, and incubated for 20 min at room temperature prior to adding to HEK 293T cells. The following day, media of HEK 293T was changed to Cnt-Prime or Epilife medium supplemented with 5% FCS, for infection of primary keratinocytes or N/TERT-2G, respectively. The next day virus-containing supernatants were collected, filtered through a 0.45 μm syringe filter (ThermoFisher), diluted 1:3 and added to cells at approximately 50% confluency. Following incubation for 5 h at 37 °C, media was changed. Keratinocytes were selected with 1 μg/ml puromycin (Invivogen, Toulouse, France) starting three days post-infection.

### siRNA transfection

N/TERT-2G cells cultured in low-calcium FAD medium were transfected at 60% confluency using Lipofectamine RNAiMAX (ThermoFisher), Opti-MEM^TM^ and 10 nM final concentration of either Silencer® Select Negative Control No. 1 siRNA or Silencer® Select siRNA for TP63 s229400 (ThermoFisher). Media was changed 24 h post transfection to 1.5 mM calcium FAD and cells were harvested 24 h later.

### Histological and immunofluorescence staining

HEEs were fixed in 4% PFA 24 h at 4 °C then dehydrated, paraffin-embedded and cut into 4 μm sections. For histological analysis, sections from HEEs (*n* = 3 per experimental group) were dewaxed and rehydrated. Sections were stained with hematoxylin and eosin (H&E), mounted with DPX non-aqueous mounting medium (Merck) and analyzed using a Leica DM1000 microscope, Leica EC3 camera and LAS EZ software (Leica Microsystems, Wetzlar, Germany).

For immunofluorescence, following dewaxing and hydration, antigen retrieval was performed by heating sections at 95 °C in citrate buffer pH 6 for 20 min. Following cooling, samples were washed, blocked and incubated with primary antibodies (Table S4) overnight at 4 °C following manufacturer’s indications. Following washing and incubations with Alexa Fluor^TM^-conjugated secondary antibodies (Table S4) and DAPI (Invitrogen) to stain nuclei, sections were mounted using Mowiol (Calbiochem-Merck) with DABCO (Merck), and images were collected and analyzed using a Leica DM6B Thunder Imager with LAS X software or a Zeiss LSM 980 Airyscan Confocal Microscope with Zen software (Oberkochen, Germany). Tiled confocal images were generated in random locations of HEEs (n = 3 biological replicates per experimental group) to quantitate total nuclei (DAPI) and those positive for GR or P63 immunofluorescence in basal and suprabasal layers. A total of 100–200 nuclei were counted per image using Image J software.

For immunohistochemical analysis, paraffin-embedded skin biopsies from AD patients (*n* = 2) and healthy individuals (*n* = 2) used for immunostaining were dewaxed, hydrated, subjected to heat-induced antigen retrieval with citrate buffer prior to blocking the endogenous peroxidase activity with methanol: H_2_O_2_ (29:1). Sections were blocked with 5% fetal bovine serum in PBS then incubated with indicated primary and secondary antibodies (Table  S4). The avidin-biotin complex (ABC) kit (Dako, Vectastain Elite; Vector Laboratories, Inc., Burlingame, CA) was used to visualize the reaction using diaminobenzidine as chromogenic substrate for peroxidase. After hematoxylin counterstaining, slides were mounted and analyzed by light microscopy (Leica DM RXA2).

### RNA isolation, cDNA preparation and qPCR

RNA was isolated from keratinocytes using Tritidy (PanReac), the phase separation reagent 1-bromo-3-chloropropane (Merck), molecular biology grade ethanol and isopropanol (Panreac) and nuclease-free water (ThermoFisher). cDNA was generated using the RevertAid H Minus Reverse Transcriptase kit (ThermoFisher). QPCR was performed with gene-specific oligonucleotides (Table S4; Merck), the TB Green Premix Ex Taq and Rox reference dye II (Takara, Goteborg, Sweden) and the Quant Studio 5 Real-Time PCR System (Applied Biosystems, ThermoFisher). Technical triplicates were used; and 3 independent biological replicates per experimental group were assessed to calculate the mean value ± SD. For amplification of cDNA, Ct values were normalized to *RPLPO*. In the case of ChIP-QPCR, Ct values of each ChIP were normalized to those of respective inputs. Primers are in Table S4.

### Immunoblotting

Keratinocyte lysates (3 independent biological replicates per experimental group) were prepared using RIPA buffer (50 mM Tris pH 8, 150 mM NaCl, 0.5% sodium deoxycholate, 0.1% SDS, 1% IGEPAL® CA-630) or total protein was purified using Tritidy, as described [60]. Briefly, cells were lysed in Tritidy, RNA and DNA were precipitated, and the protein-containing phase was transferred to new tubes. An excess of isopropanol was added to precipitate protein and pellets were washed twice with 95% ethanol. Protein precipitates were dried, resuspended in lysis buffer (100 mM Tris pH 8, 20 mM EDTA, 140 mM NaCl, 5% SDS) supplemented with protease and phosphatase inhibitors and incubated at 50 °C for 2 h. Protein concentrations were measured using the BCA protein assay kit (Pierce, ThermoFisher) and 15–30 μg of protein/sample was boiled in Laemmli buffer, separated on SDS-PAGE, and transferred to Hybond ECL nitrocellulose (GE Healthcare, ThermoFisher). Nitrocellulose membranes were stained with Ponceau S (Merck) to verify protein loading and transfer prior to blocking in TBST-5% milk and subsequent antibody incubations. See Table S4 for details of primary and secondary antibodies used. The Pierce ECL Plus Western Blotting Substrate (ThermoFisher) and the ImageQuant 4000 Biomolecular Imager (GE Healthcare) were used to detect immunoreactive signal. Band intensities were quantitated using Image J software and were normalized to Actin or Tubulin.

### Antibody arrays

The Proteome Profiler™ Human XL Cytokine Array Kit (ARY022B; R&D Systems, Abingdon, UK) contains antibodies to 105 human cytokines, chemokines, and growth factors, and was used to evaluate protein levels in keratinocyte culture supernatants. The array contains 2 technical replicates for each antibody spot, and 3 independent biological replicates samples were assessed per experimental group. Only factors with a *p* value < 0.05 are represented in Figs. 5d and Table S3. Western Vision Software was used to quantify signal of each spot (R&D Systems).

### Statistics and data analysis

Unless otherwise mentioned, statistical significance of experimental data was calculated using the GraphPad Prism software version 8 (San Diego, CA). Data represent independently repeated experiments; in graphs, mean values ± SD are shown. Prior to parametric testing, the Levene’s test was used to determine whether samples within groups had equal variance. For comparisons between two experimental groups, we used the Student’s unpaired two-tailed t-test. For comparisons among more than two experimental groups, we used one- or two-way ANOVA, which if statistically significant was followed by a post hoc Tukey multiple comparison test. *P* values < 0.05 were considered statistically significant. Metascape was used for Gene Ontology categorization [61]; https://metascape.org/gp/index.html#/main), evaluating Biological Process or Molecular Function with a p-value set to 0.01. For enrichment analysis, Enrichr software (https://maayanlab.cloud/Enrichr/) and the ChEA3 2022 gene set library was used; Biovenn was used to generate Venn diagrams [62]. Heat maps were generated with Flaski https://flaski.age.mpg.de/).

### Supplementary information

Supplemental figures

Table S1

Table S2

Table S3

Table S4

Supplemental material

## Data Availability

The corresponding author can provide the data that supports the findings of this study upon request to any researcher wishing to use them for non-commercial purposes.
