# Supplementary material for: Glucocorticoid receptor controls atopic dermatitis inflammation via functional interactions with P63 and autocrine signaling in epidermal keratinocytes
Source: Cell Death Dis. 2024 Jul 28;15(7):535. doi: 10.1038/s41419-024-06926-w (PMC11284228; doi:10.1038/s41419-024-06926-w)
Supplement: Supplementary file 1 — Supplemental figures [file 41419_2024_6926_MOESM1_ESM.pdf]

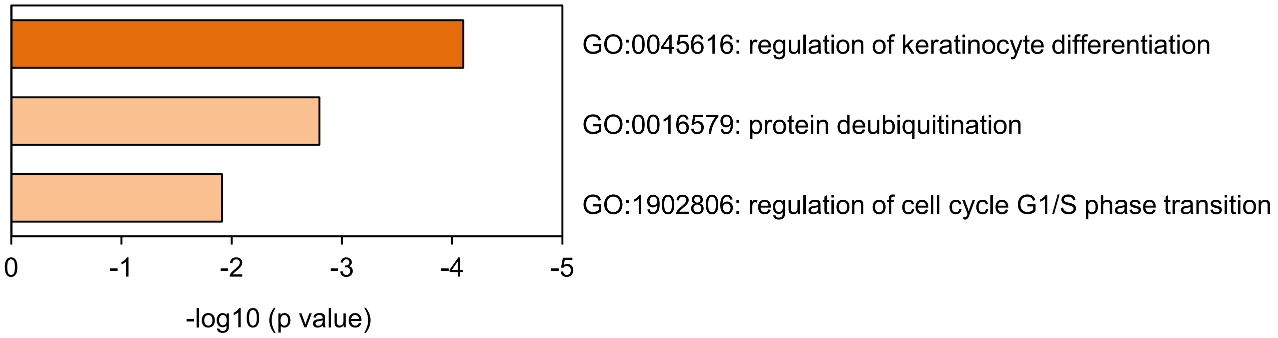

Fig. S1. Categorization of novel GR interactors identifies regulation of keratinocyte differentiation as the most overrepresented biological process

Enrichment analysis using GO Biological Process (Metascape) of 52 significant novel interactors (gain) identified regulation of keratinocyte differentiation as the most overrepresented process. Interactor proteins included TP63, GRHL1, GRHL2, KLF5, and RUNX1.

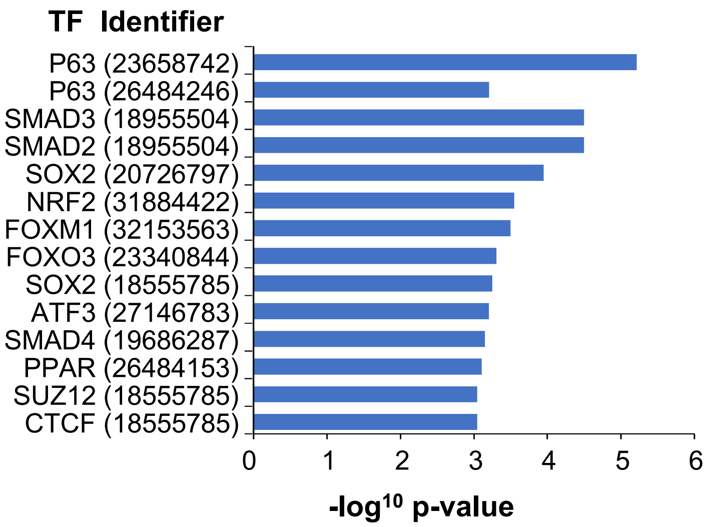

Fig. S2. Enrichment analysis to assess overlap between differentially expressed genes identified in GR^{EKO} epidermis with ChIP-Seq datasets.

Analyses were performed using Enrichr software (https://maayanlab.cloud/Enrichr/) and the ChEA3 2022 gene set library., and identified enrichment of 2 P63 ChIP-Seq datasets among those with statistically significant overlap.

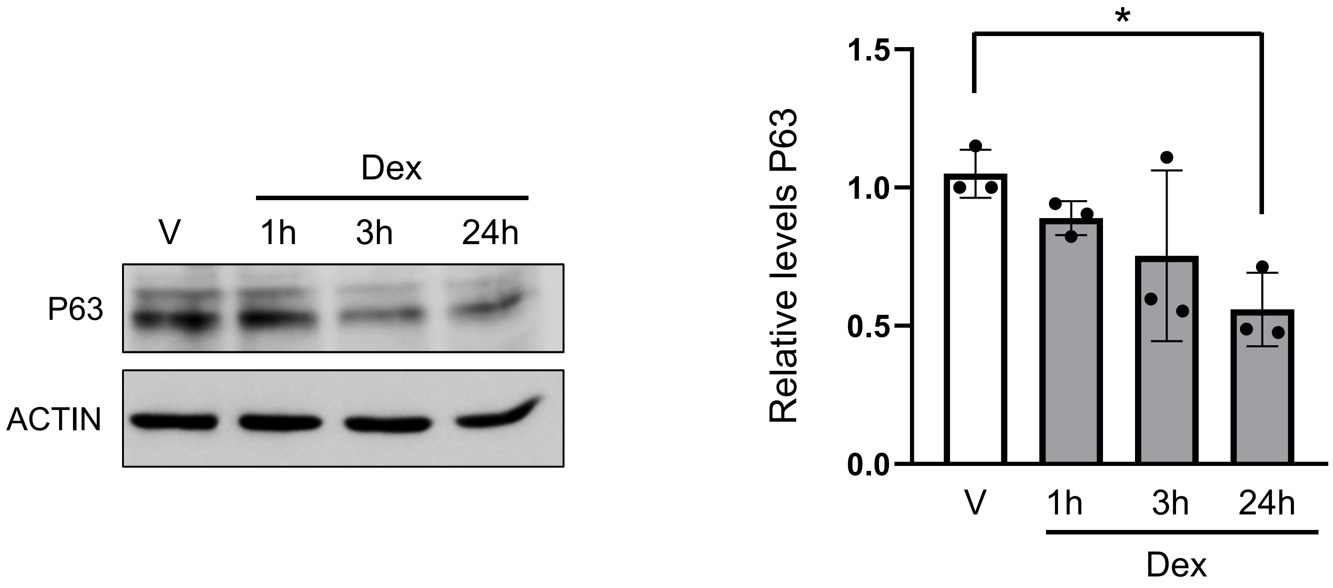

Fig. S3. P63 protein is downregulated following 24h treatment with Dex. N/TERT-2G cells were incubated with vehicle (V) or 1μM Dex for the indicated times prior to immunoblotting, left. Graph showing quantitation is on right. N= 3 for each experimental group. Statistical significance was determined using one way ANOVA and post hoc Tuckey multiple comparison test indicated as: * $p<0.05$.

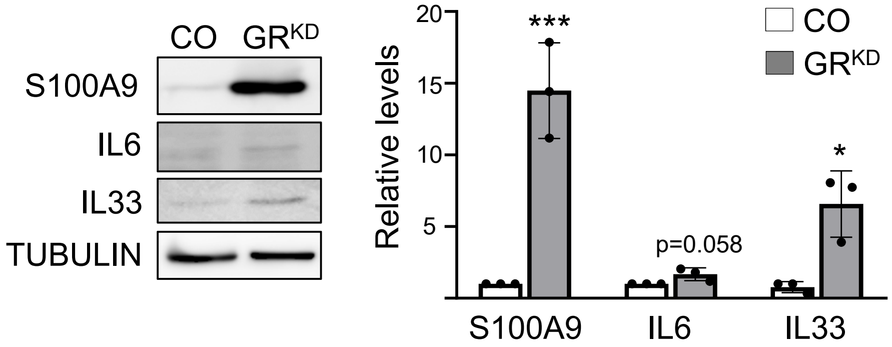

Fig. S4. Intracellular protein levels of inflammatory mediators in CO or GR^{KD} N/TERT-2G cells

Immunoblot (left) and quantitation (right) of control (CO) or GR-knockdown (GR^{KD}) keratinocyte lysates with indicated antibodies. N= 3 for each experimental group. Statistical significance was determined using Student´s t test indicated as: * $p<0.05$; *** $p<0.001$.

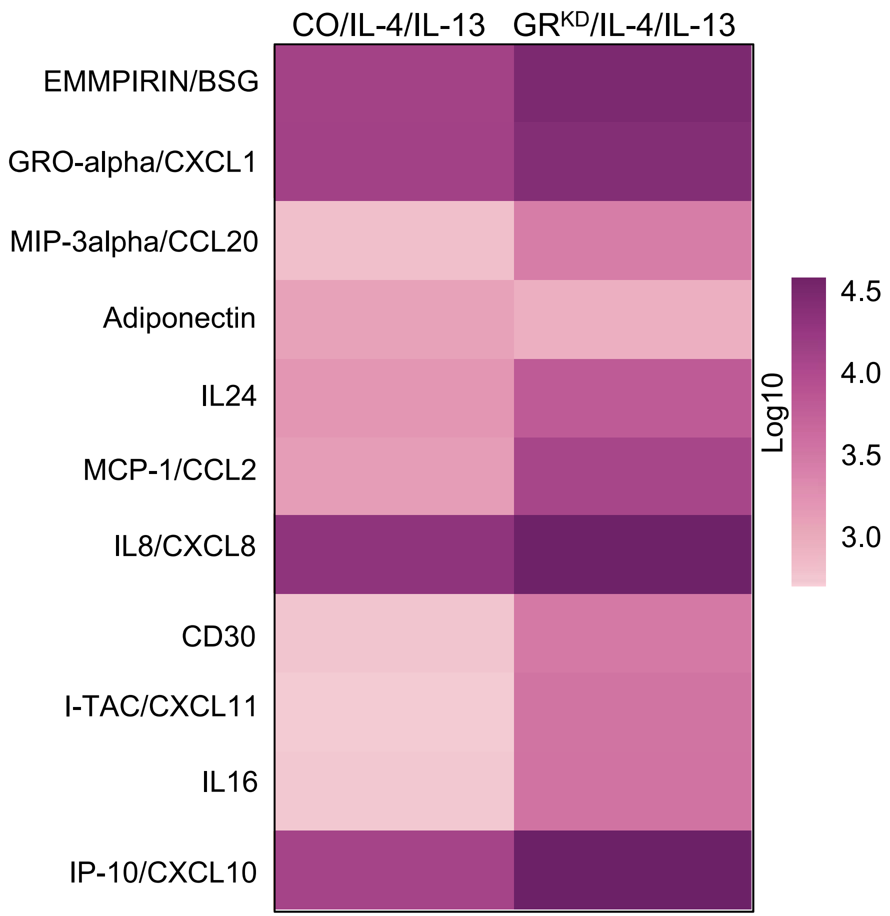

Fig. S5. Profile of secreted factors in IL4/IL13-treated CO or GR^{KD} primary keratinocytes
Heatmap shows relative levels of secreted factors in CO or GR knockdown (GR^{KD}) primary keratinocyte supernatants in the presence of IL4/IL13 (50ng/ml, 24h), using a multiplex antibody array. Data represent average of three independent experiments; all factors in the heatmap showed statistical significance using two-way ANOVA; $p < 0.05$.

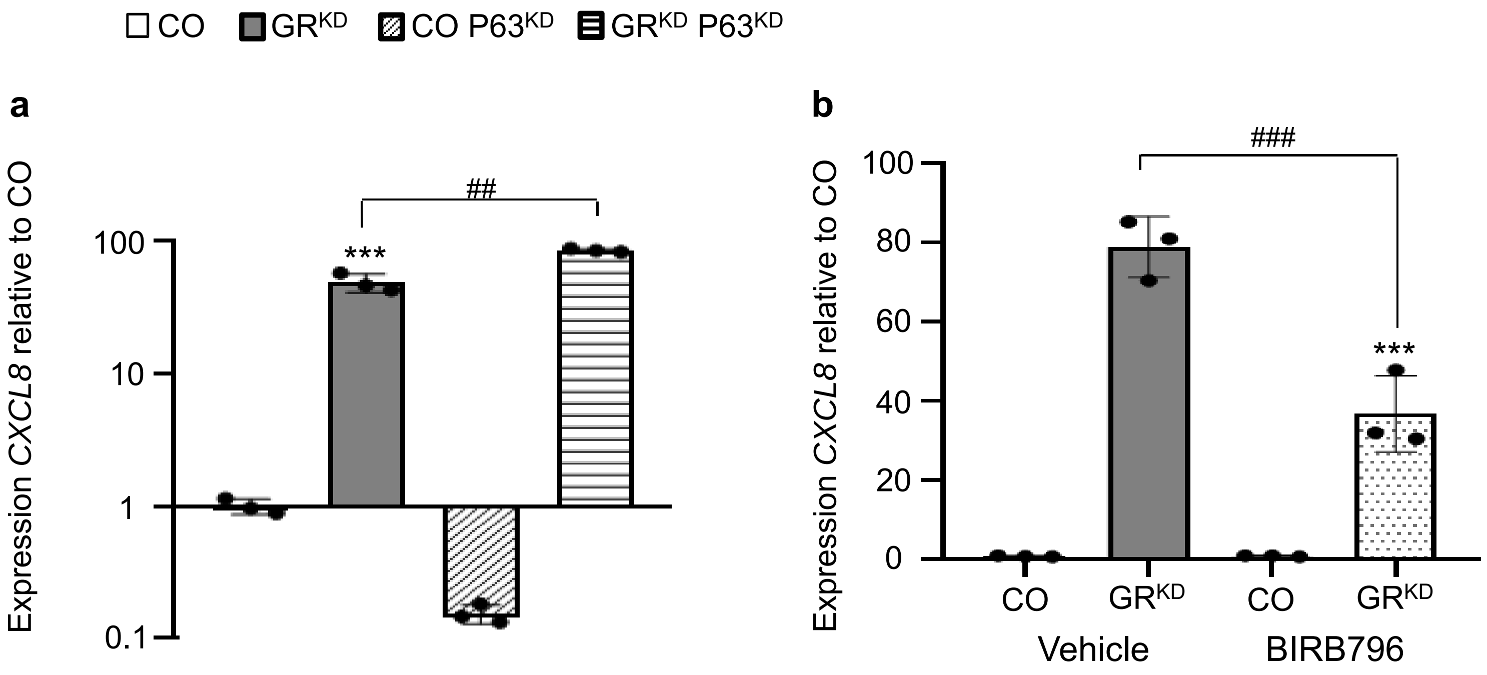

Fig. S6. Increase in *CXCL8* upon GR knockdown is dependent on P38-MAPK but not P63

a. RT-QPCR for *CXCL8* in CO or GR^{KD} N/TERT-2G cells 48h following transient transfection with negative control or P63 specific siRNA ($P63^{KD}$). While $P63^{KD}$ cells have a significant decrease in *CXCL8* expression, paradoxically the double GR^{KD} $P63^{KD}$ cells show increased levels relative to the GR^{KD} cells. **b.** RT-QPCR for *CXCL8* in CO or GR^{KD} N/TERT-2G treated for 24h with vehicle (DMSO) or 1μM BIRB796. N= 3 for each experimental group. Statistical significance using 2-way ANOVA with post hoc Tukey multiple comparison test: ## $p < 0.01$; ***, ###, $p < 0.001$. Asterisks: significant differences relative to CO; hashes: significant differences between groups indicated by brackets.
