# Supplementary figures and images for: Glucocorticoid receptor controls atopic dermatitis inflammation via functional interactions with P63 and autocrine signaling in epidermal keratinocytes
Source: Cell Death Dis. 2024 Jul 28;15(7):535. doi: 10.1038/s41419-024-06926-w (PMC11284228; doi:10.1038/s41419-024-06926-w)

Fig. 2e

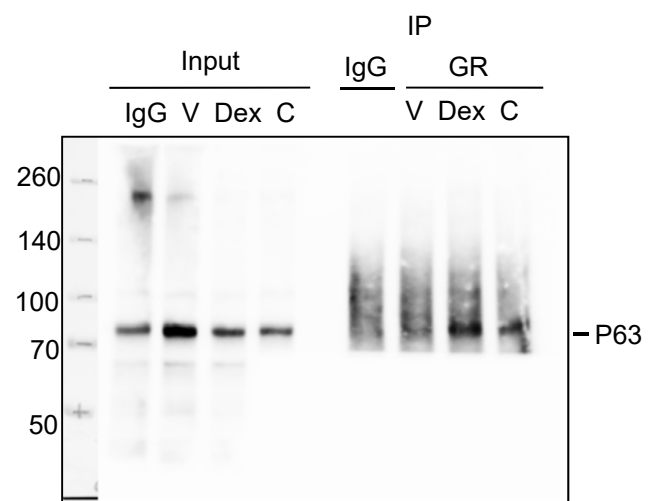

Fig. 4a

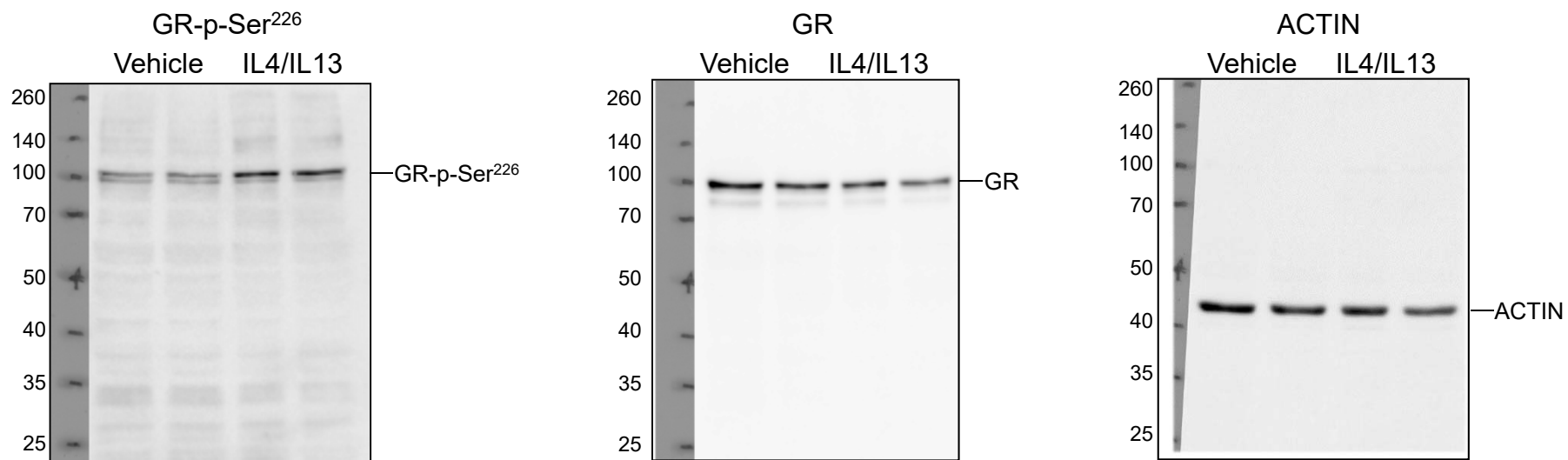

Fig. 5a

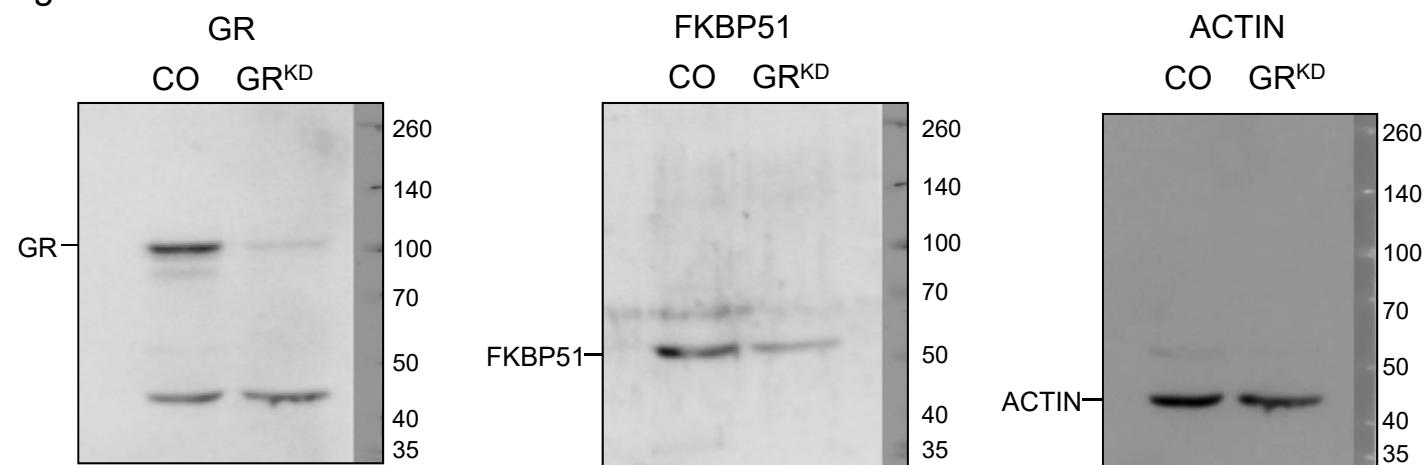

Fig. 5b

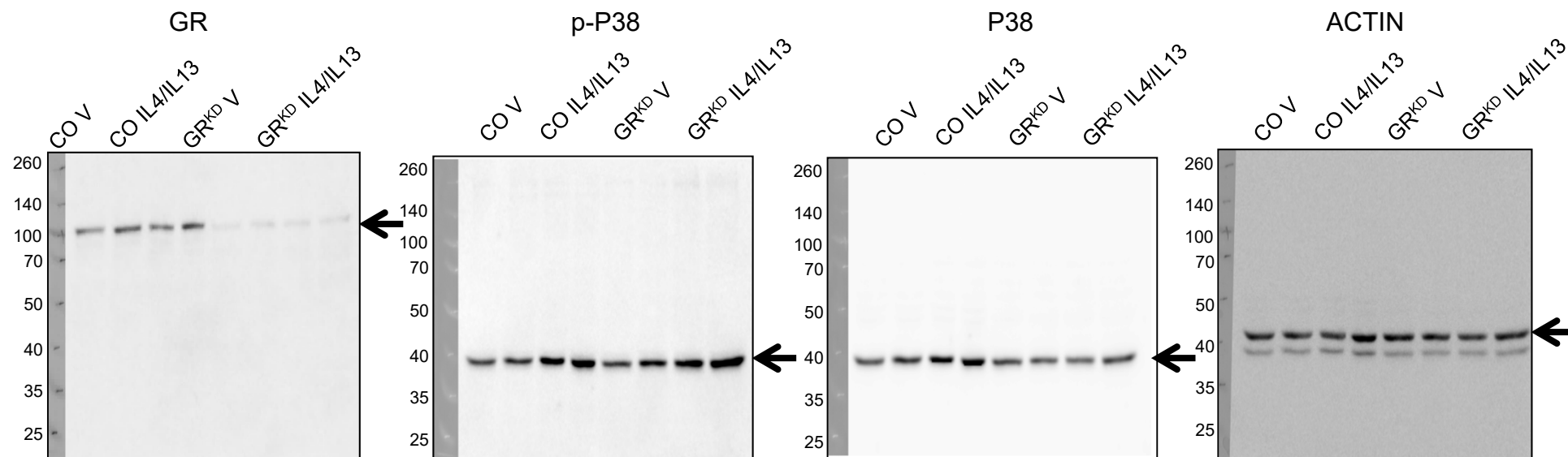

Fig. 6c

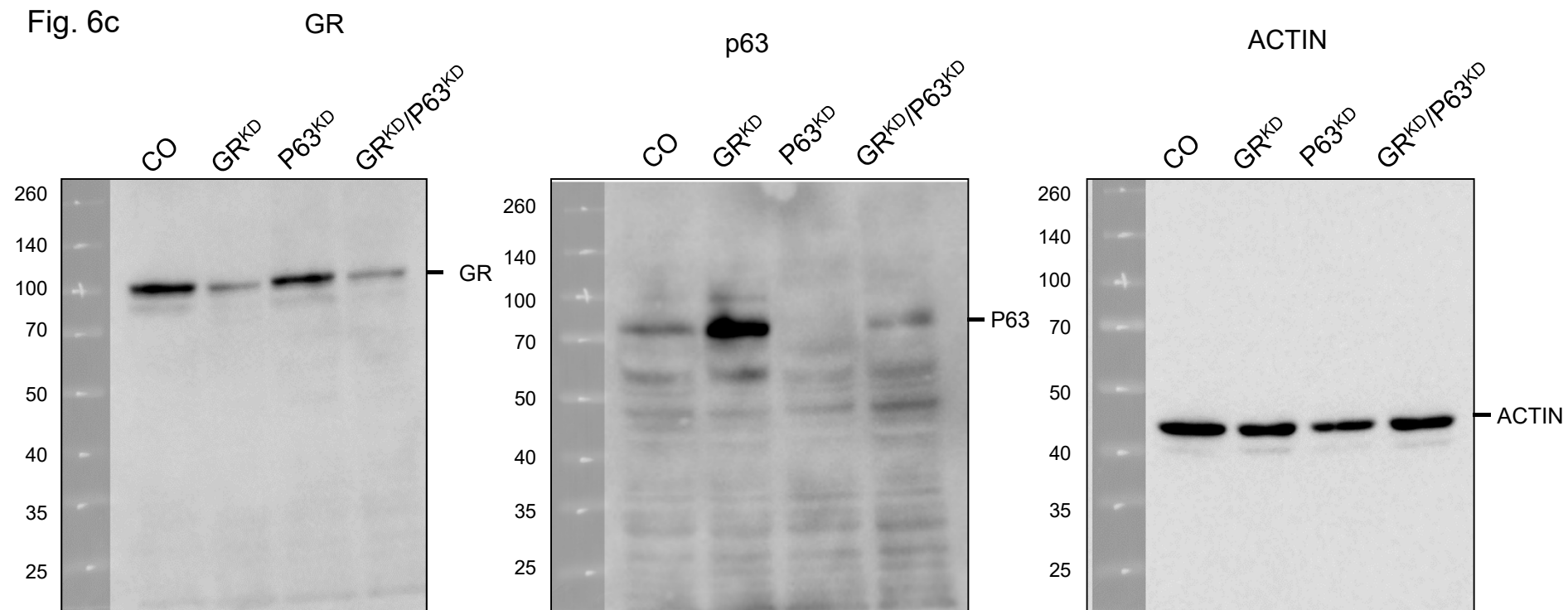

Fig. S3

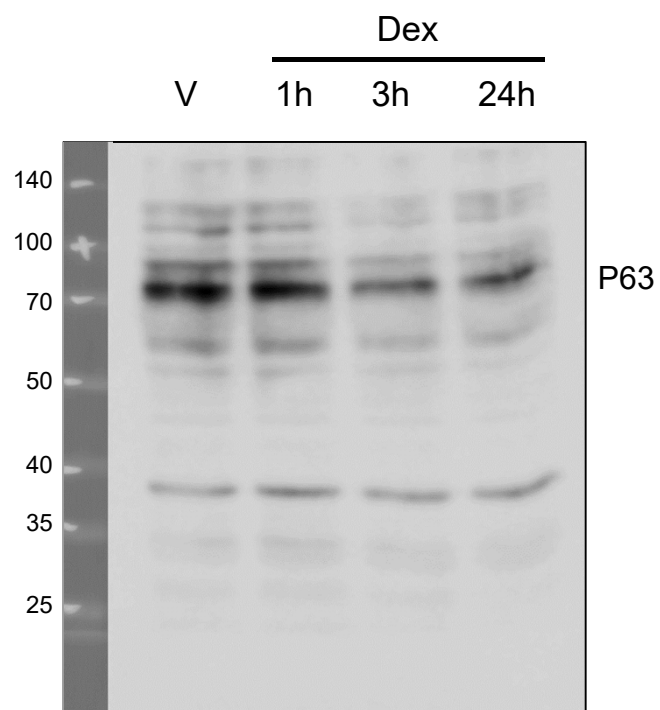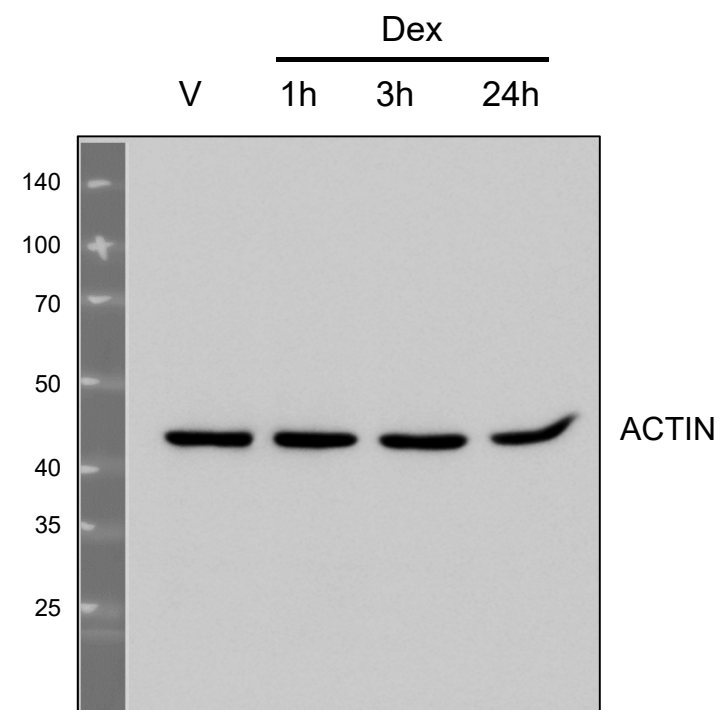

Fig. S4

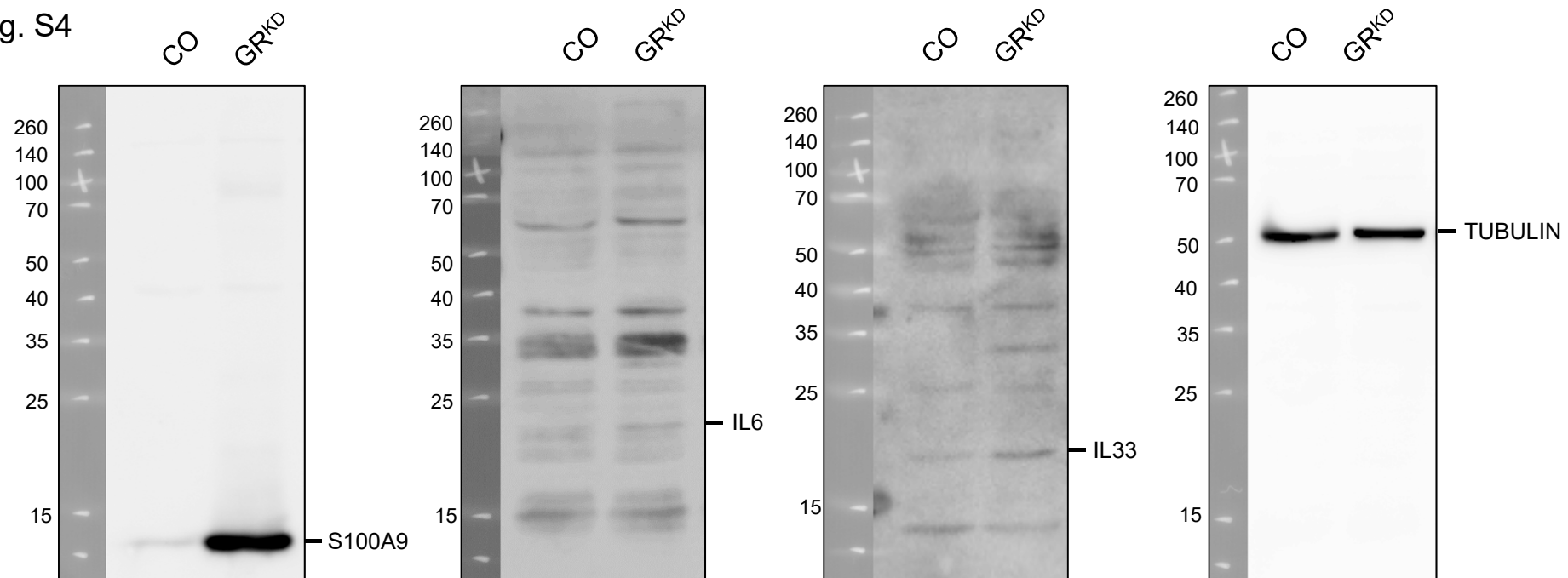

Supplement: Supplementary file 6 — Supplemental material [file 41419_2024_6926_MOESM6_ESM.pdf]
